# Toward a Biocultural Synthesis of the Peopling of the Americas: Introduction to the Special Issue

**DOI:** 10.1002/ajpa.70160

**Published:** 2025-11-11

**Authors:** Lumila Paula Menéndez, Mark Hubbe

**Affiliations:** ^1^ Department of Anthropology of the Americas University of Bonn Bonn Germany; ^2^ School of Anthropology University of Costa Rica San Jose Costa Rica; ^3^ Department of Evolutionary Biology University of Vienna Vienna Austria; ^4^ Department of Anthropology The Ohio State University Columbus Ohio USA

**Keywords:** biocultural approaches, decolonial anthropology, interdisciplinary research, peopling of the Americas, Poblamiento de las Américas, investigación interdisciplinaria, enfoques bioculturales, antropología decolonial, Povoamento das Américas, abordagens bioculturais, pesquisa interdisciplinar, antropologia decolonial

## Abstract

This article introduces the special issue *Toward a Biocultural Synthesis of the Peopling of the Americas*, which brings together contributions that explore the origins and diversity of Indigenous populations across North, Central, and South America and the Caribbean. The volume grew out of an invited symposium held at the 90th Annual Meeting of the American Association of Biological Anthropologists and reflects a shared commitment to methodological pluralism, regional specificity, and ethical collaboration. By integrating genetic, skeletal, linguistic, and archaeological data with Indigenous perspectives and regional histories, the papers assembled here challenge overly linear and homogenizing models of human dispersion. They highlight the value of explanatory dissonance—tensions between datasets, temporal scales, and epistemic traditions—as a productive resource for understanding the complexity of human history. The issue's contributions range from simulation models and morphometric analyses to isotopic reconstructions and linguistic typology, offering complementary insights into population continuity, interaction, and diversification across the Americas. Commentaries by María Nieves‐Colón and Rolando González‐José situate these studies within broader disciplinary and epistemic debates, emphasizing the need for integrative, decolonial, and collaborative approaches. Together, the articles and commentaries demonstrate that the most meaningful advances in the study of the peopling of the Americas now emerge from explicitly biocultural frameworks that link biological variation, cultural history, and ethical engagement in the production of anthropological knowledge.

## ABSTRACT IN SPANISH

Este artículo introduce el volumen especial *Hacia una síntesis biocultural del poblamiento de las Américas*, que reúne contribuciones dedicadas a explorar los orígenes y la diversidad de las poblaciones indígenas de América del Norte, Central y del Sur, así como del Caribe. El volumen surgió de un simposio invitado realizado durante la 90ª Reunión Anual de la Asociación Americana de Bioantropólogos y refleja un compromiso compartido con el pluralismo metodológico, la especificidad regional y la colaboración ética. Al integrar datos genéticos, esqueletales, lingüísticos y arqueológicos con perspectivas indígenas e historias regionales, los trabajos aquí reunidos cuestionan los modelos excesivamente lineales y homogeneizadores de la dispersión humana y subrayan el valor de la disonancia explicativa —las tensiones entre conjuntos de datos, escalas temporales y tradiciones epistémicas— como un recurso productivo para comprender la complejidad de la historia humana. Las contribuciones del volumen abarcan desde modelos de simulación y análisis morfométricos hasta reconstrucciones isotópicas y tipología lingüística, ofreciendo perspectivas complementarias sobre la continuidad, interacción y diversificación poblacional en las Américas. Los comentarios de María Nieves‐Colón y Rolando González‐José sitúan estos estudios en debates disciplinarios y epistémicos más amplios, destacando la necesidad de enfoques integradores, decoloniales y colaborativos. En conjunto, los artículos y comentarios demuestran que los avances más significativos en el estudio del poblamiento de las Américas surgen hoy de marcos explícitamente bioculturales que vinculan la variación biológica, la historia cultural y el compromiso ético en la producción del conocimiento antropológico.

## ABSTRACT IN PORTUGUESE

Este artigo introduz o volume especial *Rumo a uma síntese biocultural do povoamento das Américas*, que reúne contribuições dedicadas a explorar as origens e a diversidade de populações indígenas da América do Norte, Central e do Sul, assim como do Caribe. O volume surgiu de um simpósio organizado durante a 90ª Reunião Anual da Associação Americana de Bioantropólogos e reflete um compromisso compartilhado em pluralismo metodológico, especificidade regional e colaboração ética. Ao integrar dados genéticos, esqueletais, lingüísticos e arqueológicos com perspectivas indígenas e histórias regionais, os artigos reunidos neste volume questionam modelos excessivamente lineares e homogeneizadores da dispersão humana, e enfatizam o valor da dissonância explicativa—as tensões entre conjuntos de dados, escalas temporais e tradições epistêmicas—como um recurso produtivo para se compreender a complexidade da história humana. As contribuições do volume abrangem desde modelos de simulação e análises morfométricas até reconstruções isotópicas e tipologias lingüísticas, oferecendo perspectivas complementares sobre a continuidade, interação e diversificação populacional nas Américas. Os comentários de María Nieves‐Colón e de Rolando González‐José situam estes estudos em debates disciplinares e epistêmicos mais amplos, destacando a necessidade de enfoques integradores, decoloniais e colaborativos. Em conjunto, os artigos e comentários demonstram que os avanços mais significativos no estudodo povoamento das Américas surgem atualmente de marcos explicitamente bioculturais que vinculam a variação biológica, a história cultural e o compromisso ético na produção de conhecimento antropológico.

## 1 Rethinking the Peopling of the Americas Through Interdisciplinary and Biocultural Lenses

The peopling of the Americas remains one of the most complex and debated chapters in human evolutionary history. Over the past two decades, rapid advances in genetics, archaeology, bioanthropology, and linguistics have greatly expanded our understanding of the processes that shaped the initial human dispersion into the continents and the subsequent diversification of Native American populations (e.g., Reich et al. 2012; Posth et al. 2018; Dillehay et al. 2015; Nichols 2025; see also Menéndez 2024). Yet, as the scientific disciplines dedicated to the topic become increasingly specialized, the need for integrated approaches that explicitly incorporate distinct voices and perspectives has become more urgent (Menéndez et al. 2022). The human occupation of the American continents was a complex process, as demonstrated by decades of study, and we now exist at a moment when the study of its populational history can only be effectively advanced by integrating diverse scientific datasets and methods—such as skeletal, genetic, linguistic, and archaeological evidence (Goodman et al. 1988; Leatherman and Goodman 2005)—as well as by including different narratives, ontologies, and knowledge systems, particularly those of Indigenous communities (Tuhiwai Smith 2012; TallBear 2013; Nieves‐Colón et al. 2020). This fully integrated approach to study the past incorporates human history within its biocultural perspective, which acknowledges that human practices are shaped by both biological and cultural processes, and that simplifications of the processes that shaped recent human evolution have limited ability to generate valid explanatory models about the past. At the same time, a biocultural view of past human histories is inherently complex and as such interpreting them requires a collaborative, interdisciplinary framework attentive to social context, historical contingency, and the politics of knowledge production.

Yet bringing together such heterogeneous forms of evidence and perspective does not automatically lead to synthesis. It also brings into focus the frictions—between datasets, analytical scales, and epistemic traditions—that expose the limits of single‐discipline explanations. Our claim—and primary motivation for organizing the symposium that led to this special volume—is that such methodological pluralism is not simply additive—it is transformative. When genetic, morphological, linguistic, and archaeological evidence is treated as co‐equal and analyzed together, they inevitably generate tensions and dissonances in explanatory models that unsettle overly linear models of human dispersion. Rather than seeing these mismatches as problems to be resolved, we argue that they should be understood as productive signals of the complexity of human history—signals that only become visible through deliberately pluralist frameworks in the study of human evolution (Menéndez and Veigl 2025). Such a stance resonates with broader calls in the philosophy of science and anthropology to embrace explanatory dissonance not as a flaw but as a resource for building richer and more comprehensive accounts of the human past (Menéndez et al. 2022; Lewens 2012; Longino 2021).

## 2 Integrating Regions, Datasets, and Dialogs Across the Americas

**FIGURE 1:**
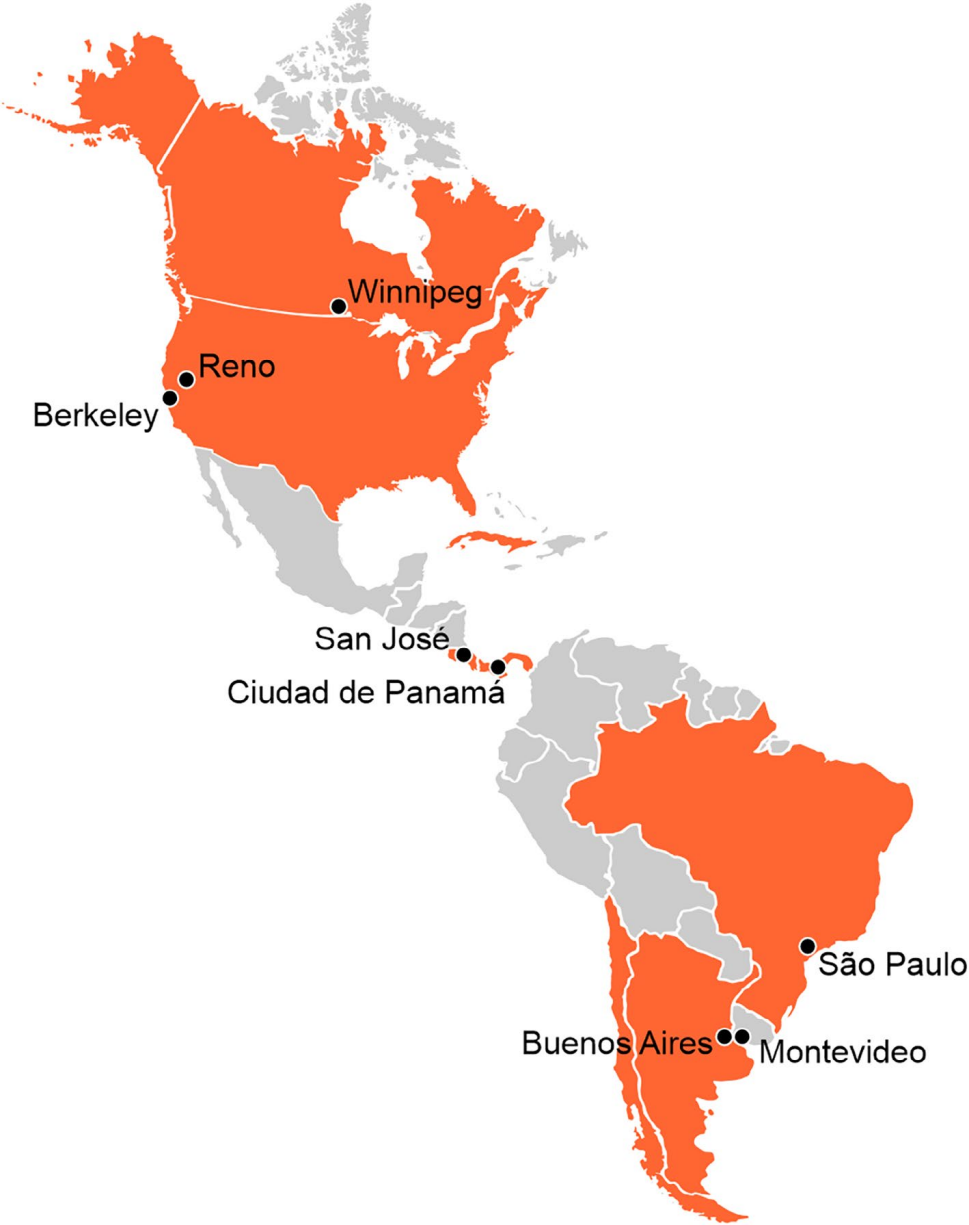
Main institutional affiliations of first authors (black dots) and geographical focus of their research (orange shading). The dots indicate the primary locations of the authors' institutions, while the shaded countries represent regions where their studies are concentrated: Argentina, Brazil, Canada, Chile, Costa Rica, Cuba, Panama and United States of America.

Responding to this call, the present special issue brings together a collection of articles that advance integrative biocultural perspectives on the origins and diversity of Indigenous populations in the Americas. The issue grew out of an invited symposium we organized for the 90th Annual Meeting of the American Association of Biological Anthropologists, which was held virtually in April 2021. By convening scholars from across different regions of the Americas—from Berkeley, Reno, and Winnipeg in North America; to San José and Ciudad de Panamá in Central America; to São Paulo, Montevideo, and Buenos Aires in South America (Figure 1)—we sought to foster dialog across diverse regional and methodological traditions. Drawing on a wide range of datasets, including skeletal, genetic, isotopic, archaeological, and linguistic evidence. The contributions collected in this volume are grounded in empirical data from regions across the hemisphere, encompassing the Arctic and sub‐Arctic, North and Central America, the Caribbean, and the Andean and Southern Cone regions of South America (Figure 1). This broad geographic and methodological scope reflects a shared commitment to building more inclusive and multivocal narratives about the human past, and takes a step toward building a more nuanced and inclusive account of population history and biocultural diversification across the American continents.

The guiding idea behind both the symposium and this special issue is rooted in the shared recognition that long‐standing models—often based on single lines of evidence and continental‐scale generalizations—fail to capture the complexity and diversity of human experiences across the Americas. In contrast, the contributions gathered here foreground regional dynamics, local contexts, and methodological pluralism. As articulated in a recent set of cross‐disciplinary recommendations (Menéndez et al. 2022), our aim is to encourage approaches that: (1) integrate multiple kinds of data from the outset, (2) remain transparent about the epistemic limits and assumptions of each method, and (3) engage with Indigenous perspectives and regional histories in order to move beyond overly linear and reductive narratives. The articles in this issue reflect this commitment in both scope and methodology. They explore genetic, cranial, linguistic, and dental variation; revisit classical models in light of new genomic or morphometric data; and challenge the assumptions embedded in continental maps marked by sweeping unidirectional arrows of migration (Hubbe et al. 2025).

Several contributions introduce novel methodological frameworks or reevaluate long‐standing theories through a biocultural lens. Figueiro's simulation‐based study explores how kinship systems and postmarital residence rules shape mitochondrial DNA diversity in archaeological contexts. Instead of treating cultural practices as background for genetic variation, it shows that social organization actively configures molecular signals. By embedding paleogenomics in anthropological theory, the study destabilizes the notion that genetic data “speak for themselves” and points toward richer, more socially grounded interpretations of genetic structure (Figueiro 2025). Castro e Silva and Hünemeier (2025) explore the biological diversity of the Tupi‐speaking people in South America and contextualize its origins within an archeologically and historically informed framework. Their review demonstrates the importance of exploring not only the Tupi expansion but also their interactions with other Indigenous and non‐Indigenous groups in South America, highlighting the complex biocultural network that integrates the Tupi and shapes their biological and cultural history. Nichols reconsiders the early linguistic landscape of North America through structural typology and founder‐effect reasoning. Her analysis reveals linguistic diversification not as a single founding event but as a multi‐stratum process shaped by ecological corridors and entry routes. By aligning linguistic features with paleoclimatic and archaeological evidence, she provides an independent yet convergent line of reasoning that both supports and complicates genetic models. This shows how language can preserve traces of population history that molecular data cannot fully capture, while also unsettling overly uniform accounts of linguistic ancestry (Nichols 2025).

Other papers in the issue highlight the value of explicitly integrating multiple types of data to reconstruct population histories in specific regions. Menéndez and Urban present a unique triangulation of craniometric, genetic, and linguistic distances among five Indigenous groups from South America's Southern Cone. Their results reveal asynchronous evolution across datasets, with each line of evidence reflecting different temporal dynamics. Instead of collapsing these differences into a single narrative, they highlight dissonances as informative in themselves—illustrating how methodological pluralism can transform our understanding of past human diversity (Menéndez and Urban 2025). In a similar vein, Scott and collaborators apply the rASUDAS2 classification system to individual‐level dental morphological data from across the Americas and Asia. Their study reaffirms a deep East Asian ancestry for all Native American groups but also reveals regionally specific affinities that undermine single‐wave migration models. By highlighting variation at continental, regional, and local scales, their analysis challenges homogenizing frameworks and underscores the interpretive limits of reductionist scenarios (Scott et al. 2025). Together, these contributions remind us that explanatory tensions across datasets are not obstacles but opportunities for richer reconstructions.

Reinforcing the commitment to regional specificity and the decentralization of North American narratives, several contributions foreground underrepresented areas of the Americas. Arencibia et al. (2025) use ancient genomic data from individuals in the Southern Cone to investigate population continuity and transformation in Patagonia. Their analysis reveals demographic processes more dynamic than previously assumed, showing that the Southern Cone cannot be reduced to a simple endpoint of continental dispersal but must be understood as a region with its own internal histories of change. In the Caribbean, Chinique de Armas and collaborators (de Chinique Armas et al. 2025) analyze isotopic data from early precolonial Cuba to reconstruct dietary diversity and mobility. Far from depicting the early Antilles as culturally homogeneous, their findings portray a mosaic of lifeways shaped by local ecologies and historical contingencies. By demonstrating that cultural diversity was present from the very beginning, their study destabilizes cultural‐historical models that treated early Caribbean populations as uniform or static. Smith‐Guzmán and collaborators (Smith‐Guzmán et al. 2025) apply dental biodistance analysis to collective burials at Cerro Juan Díaz in Panamá. Their results reveal that these burial grounds encompassed individuals from different villages and age cohorts, rather than representing extended family lineages as previously thought. This not only provides insight into mortuary practice and social organization in the Isthmo‐Colombian region but also shows how biodistance analysis can yield new perspectives in contexts where ancient DNA preservation is poor or destructive sampling is not feasible.

Together, these regionally grounded contributions challenge homogenizing frameworks and demonstrate that including areas often treated as “peripheral”—such as the Caribbean, Central America, and the Southern Cone of South America—fundamentally reshapes how we understand the diversity of past Native American populations.

Seen as a whole, these papers highlight three cross‐cutting themes. First, they underscore the importance of temporal scale: Menéndez and Urban (2025) show how different datasets evolve asynchronously, Arencibia et al. (2025) trace shifts between deep and recent continuities, Nichols (2025) demonstrates how founder effects reverberate across time in the linguistic record, and Castro e Silva and Hünemeier (2025) highlight how agricultural dispersals and language spread unfolded gradually over extended timescales in South America. Second, they decentralize the field by foregrounding regions often considered peripheral—such as the Southern Cone (Arencibia et al. 2025; Menéndez and Urban 2025; Castro e Silva and Hünemeier 2025), the Caribbean (de Chinique Armas et al. 2025), and Central America (Smith‐Guzmán et al. 2025)—showing that these regions are key to rethinking continental narratives. Third, they exemplify methodological innovation, whether through simulation modeling (Figueiro 2025), linguistic typology aligned with paleoclimate (Nichols 2025), forensic‐inspired statistical approaches to dental morphology (Scott et al. 2025), or isotopic reconstructions of diet and mobility (de Chinique Armas et al. 2025). These thematic threads underscore that the issue is more than the sum of its parts: it provides a framework for pluralist and regionally grounded approaches to human history in the Americas.

## 3 Toward a Collaborative and Decolonial History of the Peopling of the Americas

The commentaries by María Nieves‐Colón and Rolando González‐José situate the contributions of this issue within broader disciplinary and epistemic debates, highlighting that building a collaborative understanding of the peopling of the Americas requires embracing tensions across perspectives and datasets. More than reflections on the volume, they extend its central analytical claim: that apparent contradictions—such as the tension between Scott's single‐wave scenario and Nichols' multi‐stratum model—should be treated not as flaws to be reconciled but as productive signals of complexity. This recognition reinforces the idea that explanatory dissonance is a strength of biocultural and pluralist approaches, revealing dimensions of human history that would remain invisible if approached through a single data source.

Nieves‐Colón (2025) emphasizes how the articles collectively revitalize “classic” lines of evidence—linguistics, cranial and dental morphology, kinship studies, mitochondrial DNA—by reframing them with interdisciplinary perspectives, new data, and updated analytical tools. In highlighting this renewal, she shows that the contributions in this issue reject overly simplistic, linear models in favor of accounts that foreground regional variation and historical contingency. At the same time, her commentary insists on the continued relevance of nongenetic approaches in an era dominated by paleogenomics, underscoring the need to value osteological, linguistic, and isotopic evidence alongside molecular data. Just as importantly, she situates these advances within the shifting landscape of authorship and leadership in the field, where Latin American scholars are increasingly at the forefront. For her, this trend signals not only capacity building but also a move toward more inclusive, collaborative partnerships that challenge the long‐standing dominance of Global North perspectives.

Complementing this view, González‐José (2025) places this special issue in the longer arc of debates that followed the influential Greenberg, Turner, and Zegura (Greenberg et al. 1986) synthesis. He recalls how earlier disputes often locked disciplines into opposition—genetics against cranial morphology, for example—rather than seeking integrative scenarios. In contrast, he argues, the present collection exemplifies a broader shift toward genuinely interdisciplinary and, in some cases, transdisciplinary collaboration. Drawing on conceptual frameworks developed in other fields, he stresses the importance of designing projects that are integrative from the outset: where research questions, hypotheses, methods, and interpretations are co‐developed across disciplines and in dialog with Indigenous communities. Such an approach, he notes, requires iterative collaboration and ethical engagement from the earliest stages, and cannot be reduced to the ad hoc combination of results after the fact.

Taken together, these commentaries reinforce the central themes of the volume: that biocultural syntheses must be historically and regionally grounded, methodologically plural, and ethically engaged. They also point forward, urging deeper commitments to interdisciplinarity, inclusivity, and Indigenous participation as we continue rethinking the peopling of the Americas. In this sense, the present volume builds on earlier interdisciplinary efforts (e.g., González‐José et al. 2008), but pushes them further by embedding biocultural analysis in explicitly pluralist and decolonial frameworks.

In sum, the articles and commentaries in this special issue demonstrate the power of a truly interdisciplinary biocultural approach—one that understands human diversity as the product of intertwined biological processes and cultural histories, and that draws on the strengths of multiple disciplines without subordinating one to another. This perspective not only enables more comprehensive reconstructions of the past but also fosters ethical and inclusive science by foregrounding the plurality of perspectives and experiences across the continent. By bringing together diverse voices, methods, and regional case studies, the issue contributes to a growing movement for a renewed, collaborative anthropology—an anthropology that transcends disciplinary silos, challenges inherited hierarchies of knowledge, and embraces the multifaceted nature of human history. Such an anthropology must be built on sustained partnerships among researchers and descendant communities, shared decision‐making, and open exchange of data, expertise, and interpretation.

To build on this momentum, the field must continue to develop projects that are collaborative from the outset, explicitly integrate Indigenous perspectives, and embrace methodological dissonance as a generative tool rather than a limitation. Future work will also be shaped by emerging tools such as paleoproteomics and machine learning, as well as by comparisons with parallel debates in regions like Oceania and Africa, where similar tensions between datasets, scales, and narratives demand pluralist, complex, decolonial approaches. Looking forward, the biocultural perspectives advanced here enrich our understanding of the deep history of Native American populations and offer a framework that can inspire integrative and biocultural approaches to human history in other regions of the world.

## Author Contributions

**Lumila Paula Menéndez:** conceptualization, investigation, writing – original draft, writing – review and editing, project administration. **Mark Hubbe:** conceptualization, investigation, writing – original draft, writing – review and editing, project administration.

## Ethics Statement

The authors have nothing to report.

## Conflicts of Interest

The authors declare no conflicts of interest.

## Data Availability

Data sharing not applicable to this article as no datasets were generated or analysed during the current study.
